# Genetic diversity and population structure analysis of bambara groundnut (*Vigna subterrenea* L) landraces using DArT SNP markers

**DOI:** 10.1371/journal.pone.0253600

**Published:** 2021-07-01

**Authors:** Charles U. Uba, Happiness O. Oselebe, Abush A. Tesfaye, Wosene G. Abtew

**Affiliations:** 1 Department of Horticulture and Plant Science, Jimma University, Jimma, Ethiopia; 2 Ebonyi State University Abakalilki, Abakalilki, Nigeria; 3 International Institute of Tropical Agriculture, Ibadan, Nigeria; National Cheng Kung University, TAIWAN

## Abstract

Understanding the genetic structure and diversity of crops facilitates progress in plant breeding. A collection of 270 bambara groundnut (*Vigna subterrenea* L) landraces sourced from different geographical regions (Nigeria/Cameroon, West, Central, Southern and East Africa) and unknown origin (sourced from United Kingdom) was used to assess genetic diversity, relationship and population structure using DArT SNP markers. The major allele frequency ranged from 0.57 for unknown origin to 0.91 for West Africa region. The total gene diversity (0.482) and Shannon diversity index (0.787) was higher in West African accessions. The genetic distance between pairs of regions varied from 0.002 to 0.028 with higher similarity between Nigeria/Cameroon-West Africa accessions and East-Southern Africa accessions. The analysis of molecular variance (AMOVA) revealed 89% of genetic variation within population, 8% among regions and 3% among population. The genetic relatedness among the collections was evaluated using neighbor joining tree analysis, which grouped all the geographic regions into three major clusters. Three major subgroups of bambara groundnut were identified using the ADMIXTURE model program and confirmed by discriminant analysis of principal components (DAPC). These subgroups were West Africa, Nigeria/Cameroon and unknown origin that gave rise to sub-population one, and Central Africa was sub-population two, while Southern and East Africa were sub-population three. In general, the results of all the different analytical methods used in this study confirmed the existence of high level of diversity among the germplasm used in this study that might be utilized for future genetic improvement of bambara groundnut. The finding also provides new insight on the population structure of African bambara groundnut germplasm which will help in conservation strategy and management of the crop.

## Introduction

Bambara groundnut (*Vigna subterranea* (L.) Verdc) is an underutilized African legume, grown mostly, by smallholder and subsistence farmers in Sub-Sahara Africa [1]. It consists of *Vigna subterranea* var *subterranea* (cultivated) and *Vigna subterranea* var *spontanea* (wild), which are the two botanical forms both having the same number of chromosomes (2n = 22). It is the third most important legume in terms of consumption and production, after groundnut (*Arachis hypogaea*) and cowpea (*Vigna unguiculata*) in semi-arid Africa and plays an important role, especially during times of famine, serving as good source of nutrition and income [2]. The crop is drought tolerant, thrives in marginal soils with low soil fertility and contains a high amount of protein (18%), energy (65%), minerals (67%), fat (6%), essential (33%), and non-essential (66%) amino acids [3]. Despite all these benefits, it has not received adequate research and extension attention that this crop of high economic importance deserves.

Bambara groundnut in the genuine wild state was found in North Yola province of Nigeria near Garoua in Cameroon [4] with most of the abundant genetic resources existing in the corridor of Nigeria and Cameroon, which is believed to be its origin of dispersal [5]. The crop has a long history of cultivation, though its cultivation is still from local landraces rather than established varieties with a specific breeding purpose suited to a particular agro-ecological region or system of production [6]. In Africa, 0.3 million tonnes of annual bambara groundnut production has been reported [6–8] and half of its production in West Africa [7] with countries like Burkina Faso, Cameroon, Democratic Republic of the Congo, Mali, Niger and Togo reported to produce approximately 180 metric tones from a cultivation area of 250,000 ha annually [8]. Although, occasionally grown in Asia and elsewhere, its cultivation is rare outside the African continent [7]. Bambara groundnut had no improved and released seed varieties with better agronomic traits for small-scale and commercial production. Hence, unavailability of good quality seeds of the improved varieties, limited awareness about the importance of the crop among the growers, and lack of introduction of high yielding genotypes in areas of its cultivation are among the major reasons for its low production and productivity [9]. Furthermore, inadequate knowledge on the taxonomy, reproductive biology coupled with the genetics of agronomic and quality traits, pest and diseases [10], lack of genetic improvement and adaptation to particular agro-ecological zones [11] are some of the constraints for developments. This has led to underutilization of the crop resulting in weak or no genetic improvement effort, lower crop yield and quality. Bambara groundnut landraces conserved *ex-situ* in international and regional gene bank is about 6,145 with International Institute of Tropical Agriculture (IITA, Ibadan) conserving the largest germplasm [11] collected from 25 African countries with highest number of accessions from the Western Africa region [4].

Bambara groundnut landraces under low input farming systems have maintained a noticeably amount of genetic diversity [12] and investigating the genetic diversity and population structure of such germplasm provides useful information for the exploitation of the genetic resource to broaden the genetic base of bambara groundnut gene pool for the purpose of selecting the best parents for further improvement. The efficient analysis of genetic diversity within landrace collections of bambara groundnut has been recognized, as highly strategic and should be the first step towards the establishment of a coordinated breeding program [13,14]. Information on genetic relationships has been used to determine genetic distance among the genotypes tested [15], which helped categorize the genotypes into different groups based on their genetic similarity to each other. This in turn is important to select genetically divergent parental lines derived from the different genetic groups [16]. Considering the use of divergent parents in crossing programs is expected to produce high heterosis in the progenies [17,18] and increase the chance of obtaining superior segregants in advanced generations and is important to enhance the genetic base. Morphological and agronomic traits that have been used most often for the study of genetic diversity are unstable because environmental variability and expression of desired traits are influenced at the developmental stages [19]. The importance of using molecular markers to assess population structure and genetic diversity within available germplasm collections of underutilized species to enable their effective utilization by breeding program has been emphasized and is gaining momentum [16,20,21]. The use of molecular markers for the analysis of genetic diversity of germplasm is considered as reliable tools [22,23] because they are independent of environmental factors and are capable of detecting differences in alleles or changes in DNA sequence [24]. Several molecular markers like RAPD [15,25], AFLP [26], SSR [14,27], silicoDArT marker [4] have been successfully used to investigate the phylogenetic relationships and the genetic diversity of bambara groundnut.

However, considering the large genetic resource of the crop globally [11], only less than 35% of these accessions have been evaluated using molecular markers. There is paucity of report on genetic diversity and population structure with SSR, AFLP, RAPD and silcoDArT using molecular markers in bambara groundnut, when compared with other major crops like soybean, rice, and wheat. Furthermore, previous bambara groundnut studies on genetic diversity have used fewer numbers of accessions, in comparison with the current study, except Rungnoi et al. [28] who used dominant markers to evaluate 363 genotypes. Dominant markers are unsuitable for detailed assessment of genetic diversity for improvement and conservation [27]. A comprehensive study with co-dominant marker and larger number of accessions will help to understand the population structure and genetic diversity of bambara groundnut germplasm. The Diversity Arrays Technology (DArT) was developed over 15 years ago to support the resolution and hasten requirements for genomics applications and molecular breeding of plants, which generate more polymorphic markers and comprehensively cover the genome [29–31]. The DArTseq platform, based on Illumina next generation sequencing, produces both dominant silicoDArT markers and co-dominant SNP markers [11,32–34]. DArTseq technology has enabled a great discovery of SNPs in a broad variety of non-model organisms and gives measures of genetic divergence and diversity within the major genetic groups that involve crop germplasm [31,35]. In bambara groundnut, 554 DArT markers were used to study genetic diversity using 40 landrace accessions, and the result suggested relatively high genetic diversity among the accessions [4]. Recently, DArTseq technology was employed to identify SNP markers for the construction of genetic map in bambara groundnut populations that helped the identification of QTL candidate gene for internode length trait using the genome of closely related crops, like adzuki bean, common bean and mung bean [36]. Hence, the study aims to investigate the genetic diversity and population structure of bambara groundnut accessions obtained from different geographic regions using DArT SNP markers.

## Materials and methods

### Plant materials

A total of 270 accessions of bambara groundnut landraces were used in the study with 262 genotypes obtained from the Gene Bank of International Institute for Tropical Agriculture (IITA), Ibadan, Nigeria and eight genotypes from Crops and Soil Science Research Farm in Bunda, Malawi (S1 Table). The genotypes were collected from five regions [West (especially, Nigeria/Cameroon), Central, Southern and East Africa] and unknown origin (germplasm sourced from United Kingdom). Grouping of African regions as sources of the accessions was based on African Union grouping of the different African countries into regions (https://au.int/en/member_states/countryprofiles2); while Cameroon and Nigeria, as a possible places of Bambara groundnut domestication, were grouped together. Among the studied accessions, 43.3% were from West Africa, followed by Southern Africa (24.8%), Nigeria/Cameroon (14.4%), Central Africa (10.7%), East Africa (5.6%) and unknown origin (1.1%).

### DNA extraction and genotyping

The bambara groundnut leaves at the greenhouse were harvested at seedling stage from three plant of each accession and pooled. The pooled leaves were put inside eppendorf tube with dry ice. The leaf tissue was stored at −80°C, until the tissue was lyophilized, then ground in tubes with stainless steel beads using a plate shaker. DNA extraction was done using Nucleomag Plant Genomic DNA extraction kit, following the manufacturer’s protocol. The quality and quantity of DNA was checked on 0.8% agarose gel. The DArTSeq complexity reduction through *PstI-TaqI* digestion of genomic DNA and ligation of barcoded adapters was done, followed by PCR amplification of adapter-ligated fragments. The library was constructed, according to Kilian et al. [34], and was sequenced using Single Read sequencing run for 77 bases using Hiseq2500. SNP markers were aligned to the drafted reference genomes of bambara groundnut [37]. DArTseq marker scoring was performed using the DArT Proprietary Limited (PL’S) proprietary SNP calling algorithms (DArTsoft 14). SNP markers were scored as “0” = reference allele homozygote, “1” = SNP allele homozygote and “2” = heterozygote.

The marker quality was evaluated based on the individual marker related statistics, as suggested by Triticarte Pty Ltd. SNP marker with more than 80% call rate and 95% reproducibility was selected and non-polymorphic markers i.e., having a variance close to 0 with unknown SNP position were removed. The called SNPs were further filtered using TASSEL software [38], and SNPs with more than 20% missing data were removed, like those with minor allele frequency below 0.01. Finally, 3343 SNP markers were retained after filtering and data quality control.

### Field experiment and phenotypic data

The field experiment was conducted in the year 2019 at the Research and Experimental Farm of Jimma University, College of Agriculture and Veterinary Medicine at Ela-Dale. The site is located in the Southwestern part of Ethiopia, in Oromia Regional State, which is 356 km Southwest of Addis Ababa. It is classified as mid-altitude sub humid zone (7° 42 N latitude and 36° 50 E longitude) and has an altitude of 1710 masl. It receives an average annual rainfall of 1250 mm, and has an average maximum temperature of 26.2°C. The average soil pH of the farm is in the range of 5 to 6.0 [39]. The experimental design used was alpha lattice design with two replications. Two seeds were planted per hill at a depth of 5 cm with inter and intra row spacing of 50 cm x 30 cm, respectively, and later thinned to one seed per hill after emergence. Other crop management practices were carried out according to the best practices recommended for bambara groundnut production. Twelve agro-morphological traits (days to 50% flowering, days to maturity, petiole length, terminal leaflet length, terminal leaflet width, plant height, number of pods per plant, pod length, pod width, pod dry weight and seed weight per plant) were selected and measured according to the descriptors established for Bambara groundnut [40].

### Statistical analysis

Descriptive statistics, phenotypic coefficient of variation, genotypic coefficient of variation and ANOVA were analyzed for the morphological traits using R version 3.4.6 [41]. Major allele frequency, mean gene diversity within population (Hs), total gene diversity (Ht), Shannon index were computed using the R package “adegenet” [42] and “hierfstat” [43]. The pairwise Nei’s [44] D genetic distances between the populations were calculated using dartR package in R software [45]. Principal coordinate axis (PCoA) was also conducted using dartR package in R [45] to determine the contributions of each component to the variation that existed in the germplasm. AMOVA was performed to quantify genetic variation at three different hierarchical levels: among regions, among populations and within populations, using *Genalex* ver. 6.502 [46], with statistical significance based on 999 permutations. The DARwin 6 software [47] was employed to construct the neighbor-joining trees based on pair-wise genetic distances among genotypes to show the relationship between the samples using 1000 bootstrap replicates.

Population structure, based on minimum cross entropy, was determined using the ADMIXTURE model program in R [48]. The percentage membership of each of the accessions to a subpopulation was assessed, assuming hypothetical subpopulations (K) ranging from 1 to 10. The optimum number of K was estimated using Bayesian Information Criterion (BIC) in adegenet package in R [49]. Population structure was further examined with discriminant analysis of principal components (DAPC) using the “adegenet” package in R statistical package [50]. Comparison of the means of all the morphological traits across the subpopulations identified was performed with boxplots constructions on R package “ggpubr” and Kruskal-Wallis test [51].

## Results

### SNP markers quality and diversity

**Fig 1:**
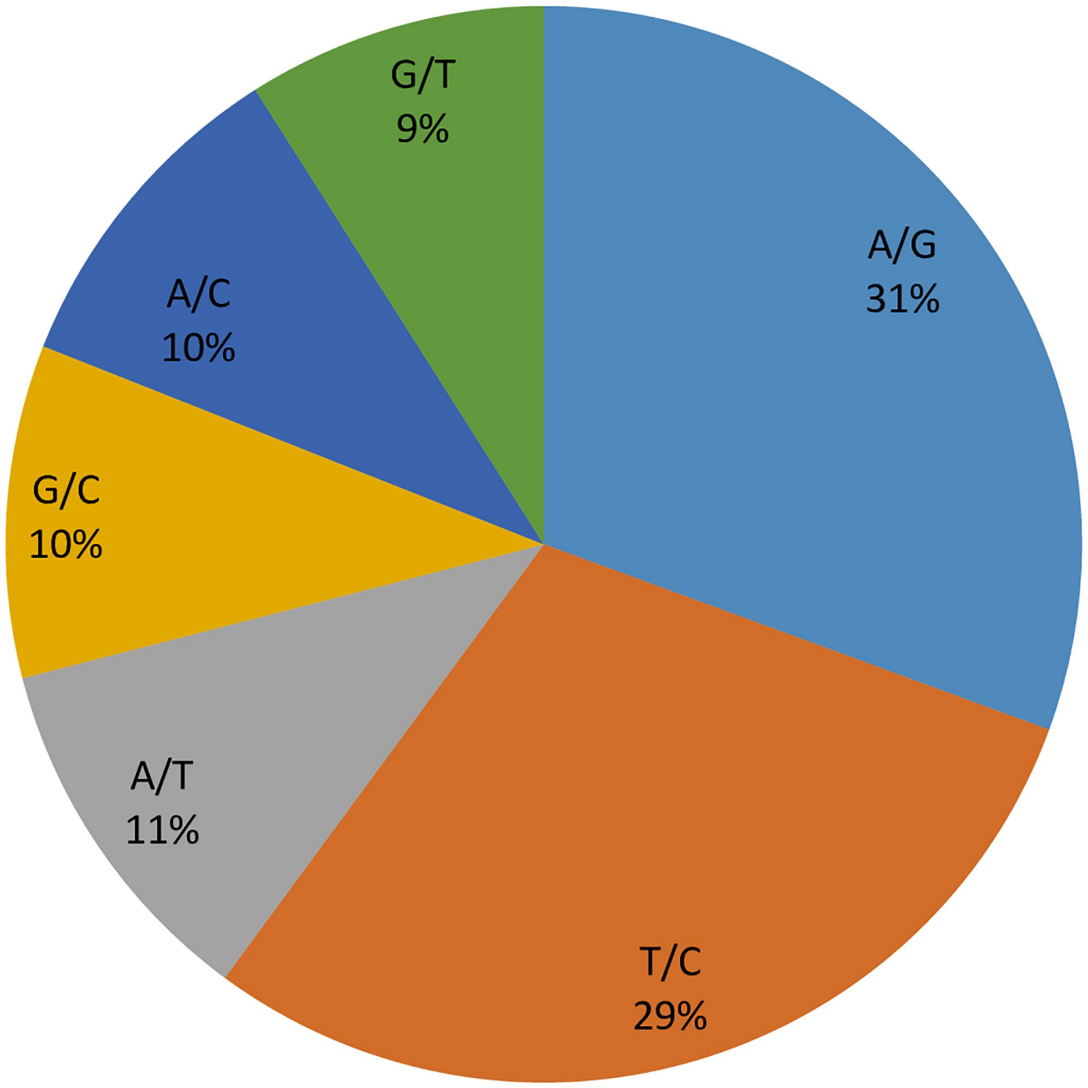
SNP type for 3334 SNP markers used in the bambara groundnut accession analysis.

The reproducibility and call rate for the mean of the 3343 SNP markers used in the study were 0.99 and 0.85, respectively, showing consistent marker score and high reproducibility (S2 Table, Table 1). The marker diversity had a mean of 0.80 for major allele frequency and 0.20 for minor allele frequency. The frequency of the two transitions (A/G, C/T) were similar with A/G having the highest frequency of 31%; while the lowest frequency among the six alleles combination was G/T with 9% (Fig 1). The frequencies of the four transversion types were 11%, 10%, 10%, 9% for A/T, G/C, A/C and G/T, respectively.

**Table 1:** Estimation of DArT SNP marker quality and diversity used for the analysis.

Marker quality and diversity	Mean	Minimum	Maximum
Major Allele frequency	0.80	0.50	0.99
Minor allele frequency	0.20	0.01	0.50
Call rate	0.85	0.80	1.00
Reproducibility	0.99	0.95	1.00
One ratio reference allele	0.26	0.02	0.50
One ratio SNP allele	0.27	0.02	0.50

### Genetic diversity

Genetic diversity was estimated per geographical region of the source of the studied germplasm (Table 2). The West Africa region had the highest mean of major allele frequency (0.91), followed by the Southern Africa (0.8), Nigeria/Cameroon (0.75), Central Africa (0.71), East Africa (0.67) regions and (0.57) unknown origin (germplam source from United Kingdom). The mean genetic diversity within each group/region revealed that the Nigeria/Cameroon and West Africa regions possess a comparable level of genetic diversity. Nigeria/Cameroon accessions had the highest (0.478) mean gene diversity within the populations across the five regions, while the least was found in the unknown origin (0.259). The comparison of the total gene diversity analysis showed that the West African region (0.482) revealed the highest diversity, followed by the Nigeria/Cameroon (0.479), East Africa (0.477), Central Africa (0.459), Southern Africa (0.456) regions and unknown origin (0.261). A similar results trend was obtained using the Shannon diversity index in which the West Africa region had the highest value (0.787), while the lowest was unknown origin (0.458).

**Table 2:** Estimation of major allele frequency, mean gene diversity within population, total gene diversity and Shannon diversity among bambara groundnut germplasm.

Region	No. of Genotypes	Major allele Frequency	Hs	Ht	I
Nigeria/Cameroon	39	0.75	0.478	0.479	0.746
West Africa	117	0.91	0.476	0.482	0.787
Central Africa	28	0.71	0.453	0.459	0.746
Southern Africa	67	0.80	0.455	0.456	0.776
East Africa	14	0.67	0.467	0.477	0.765
Unknown origin	3	0.57	0.259	0.261	0.458
Mean		0.74	0.431	0.436	0.713

where H_s_ = mean gene diversity within population, H_t_ = Total gene diversity, I = Shannon diversity.

### Genetic distance

The average Nei genetic distances among the accessions within each population revealed that the Nigeria/Cameroon and West African region had the smallest genetic distance value (0.002) (Table 3). A similar result of close relationship was observed between Southern and Eastern Africa (0.004) accessions. Central Africa accessions showed closer relationship with the Nigerian/Cameroon (0.007), while the greatest genetic distance was found between the Southern Africa and unknown origin (germplasm sourced from United Kingdom) populations (0.028). The unknown origin germplasm sourced from the United Kingdom showed a relatively distant relationship with the rest of the populations. Among the studied populations, the unknown origin population had a relatively closest relationship with the Western Africa populations (0.018), followed by the Nigeria/Cameroon populations (0.020).

**Table 3:** Pairwise Nei’s genetic distance among Bambara groundnut populations from the geographical regions.

Regions	West Africa	Nigeria/ Cameroon	Central Africa	Southern Africa	East Africa
Nigeria/Cameroon	0.002				
Central Africa	0.009	0.007			
Southern Africa	0.010	0.010	0.009		
East Africa	0.009	0.008	0.007	0.004	
Unknown origin	0.018	0.020	0.026	0.028	0.025

### Genetic differentiation

The AMOVA result showed significant genetic differences within populations (Table 4). The total genetic variance found between populations from different regions is 8%; while among populations within the region is 3%, which was the lowest variance. However, within individual populations contributed to 89% of the genetic variance. All of the three levels contributed to the overall genetic variation, as determined by the permutation analyses.

**Table 4:** Analysis of molecular variance (AMOVA) for the five geographical regions of the bambara groundnut accession.

Source	Degree of Freedom	Sum of Square	Mean Sum of Square	Estimated Variance	Percentage Variation
Among Regions	6	17799.444	2966.574	61.187	8%
Among Populations	14	14045.728	1003.266	22.369	3%
Within Populations	249	179047.801	719.067	719.067	89%
Total	269	210892.974		802.623	100%

### Principal coordinate analysis

**Fig 2:**
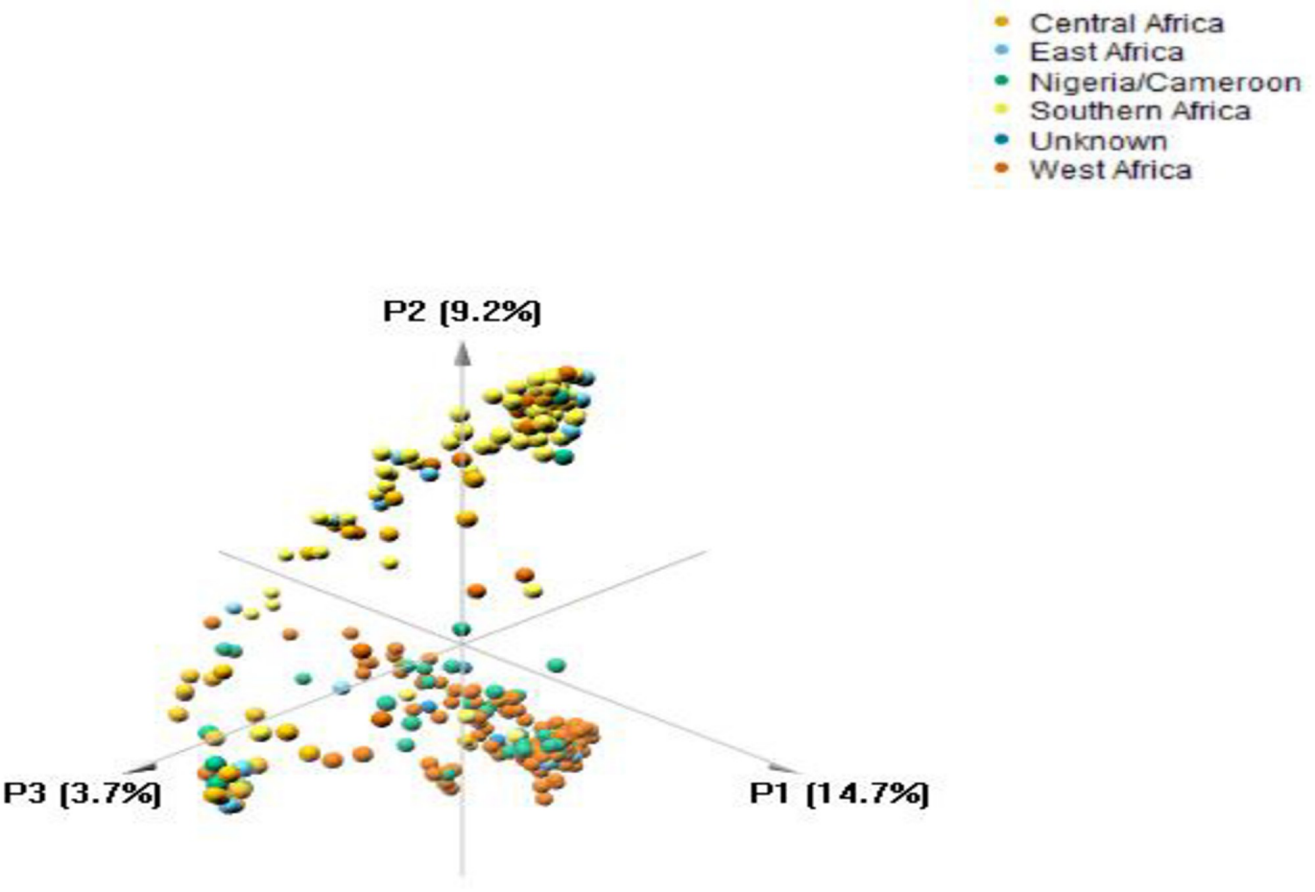
Percentage of variation explained by the first three axes.

**Fig 3:**
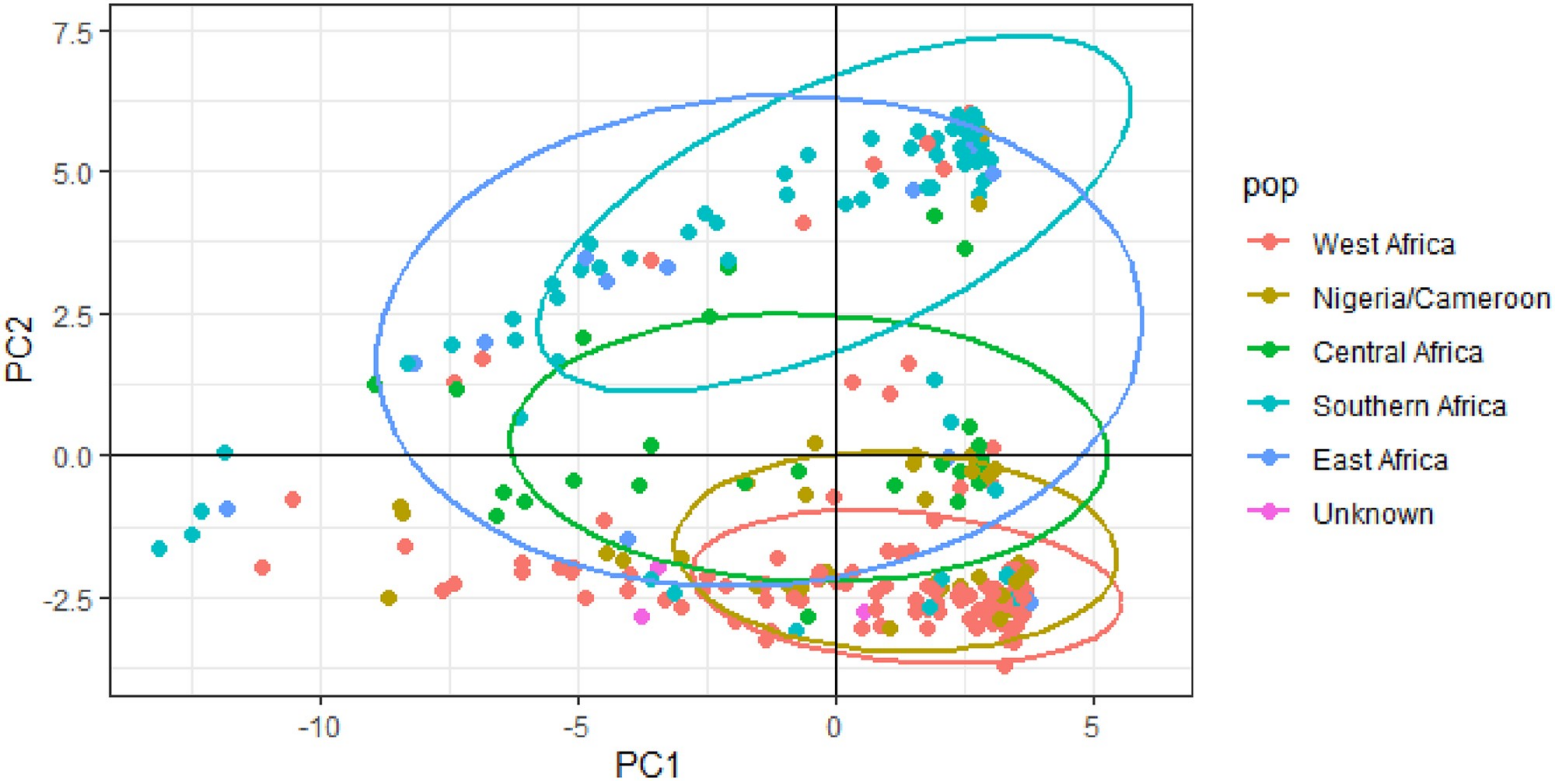
Principal component analysis showing the clustering between the geographical regions.

The cumulative percentage contributions of the first three principal components (PCs) to the total variations in the populations was 27.6%, with 14.7%, 9.2% and 3.7% respective contributions to PC1 to PC3 (Fig 2). The West Africa and Nigeria/Cameroon regions contributed most to the variation observed in PC1; while Southern and East Africa for PC2. The PCA separated the germplasm into three main groups (Fig 3). It showed a clear separation between the West Africa and Southern Africa populations, whereas there was a clear overlap between the Nigeria/Cameroon and West Africa accessions. PCA categorized the West Africa and Nigeria/Cameroon accessions into group one; whilst East and Southern Africa into group two, and the Central Africa accessions found between group one and two, into group three.

### Neighbor-joining tree analysis and discriminant analysis of principal components

**Fig 4:**
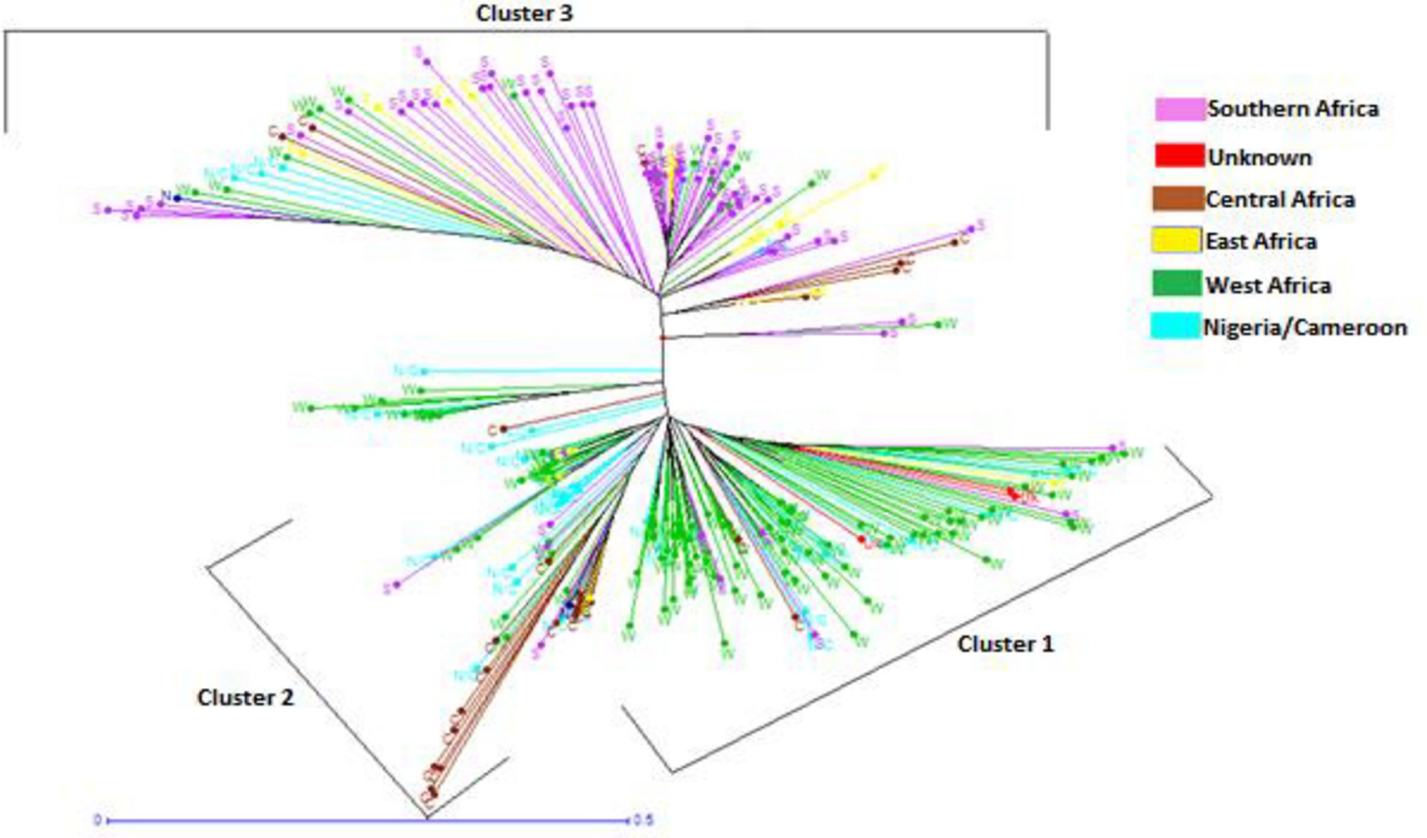
Neighbor joining (NJ) tree showing relationships among populations of bambara groundnut.

**Fig 5:**
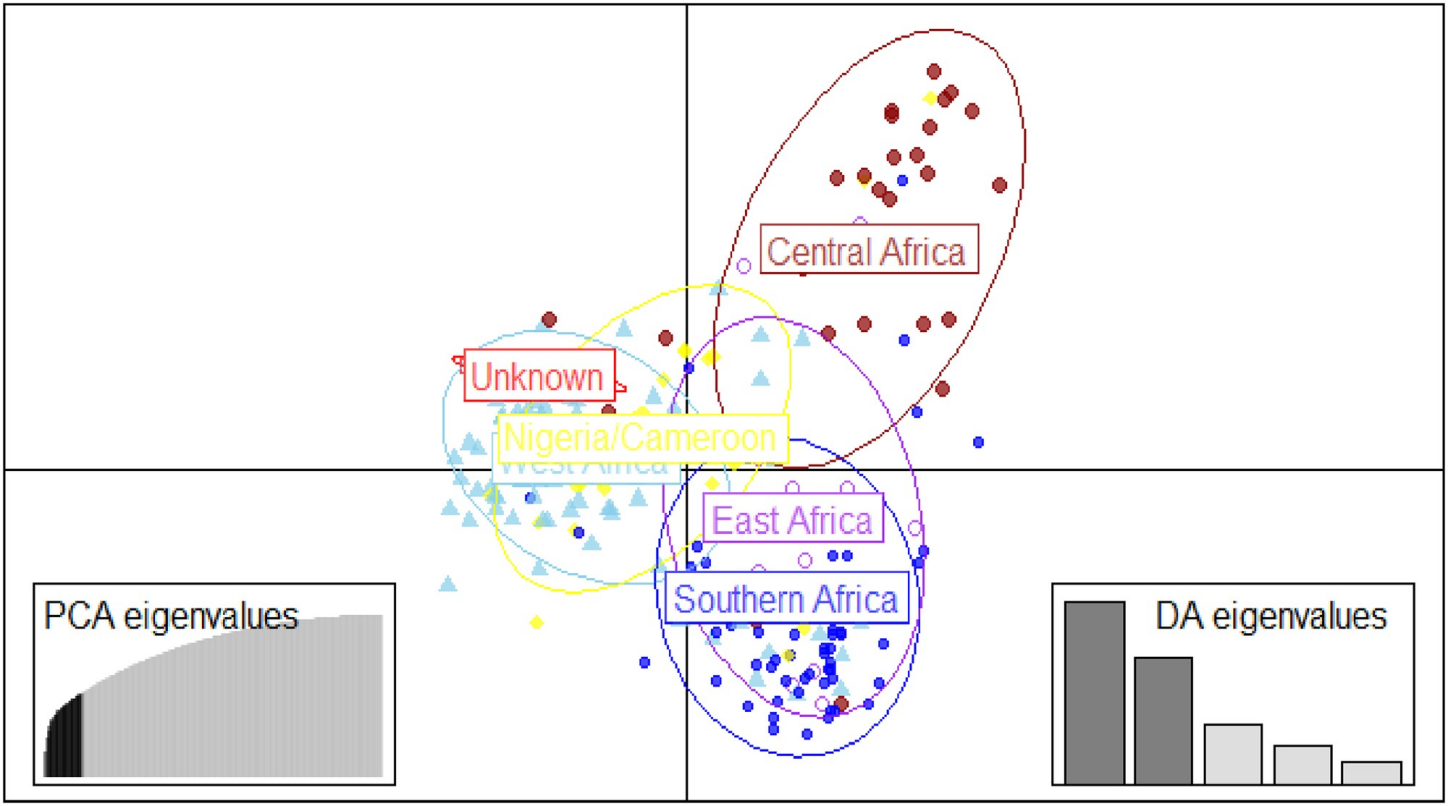
DAPC analysis showing the relationship between the geographic regions.

Neighbor-joining analysis was used to detect the genetic relationship of the bambara groundnut germplasm based on the dissimilarity matrix (S3 Table). The neighbor joining tree algorithm clearly delineated the genotypes into three major clusters (Fig 4). Some of the accessions did not group or cluster according to their geographical origin/source of their collection. All unknown origin and the majority of West African accessions were found in cluster one, while most of Central Africa accessions were found in cluster two, together with some Nigeria/Cameroon accessions. Southern and Eastern African accessions were, predominantly, in cluster three. The membership clustering of DAPC revealed three major groups of accessions (Fig 5). The West Africa, Nigeria/Cameroon and unknown origin accessions were found in one cluster; whereas the East and Southern Africa, and the Central Africa accessions were in different clusters.

### Population structure

**Fig 6:**
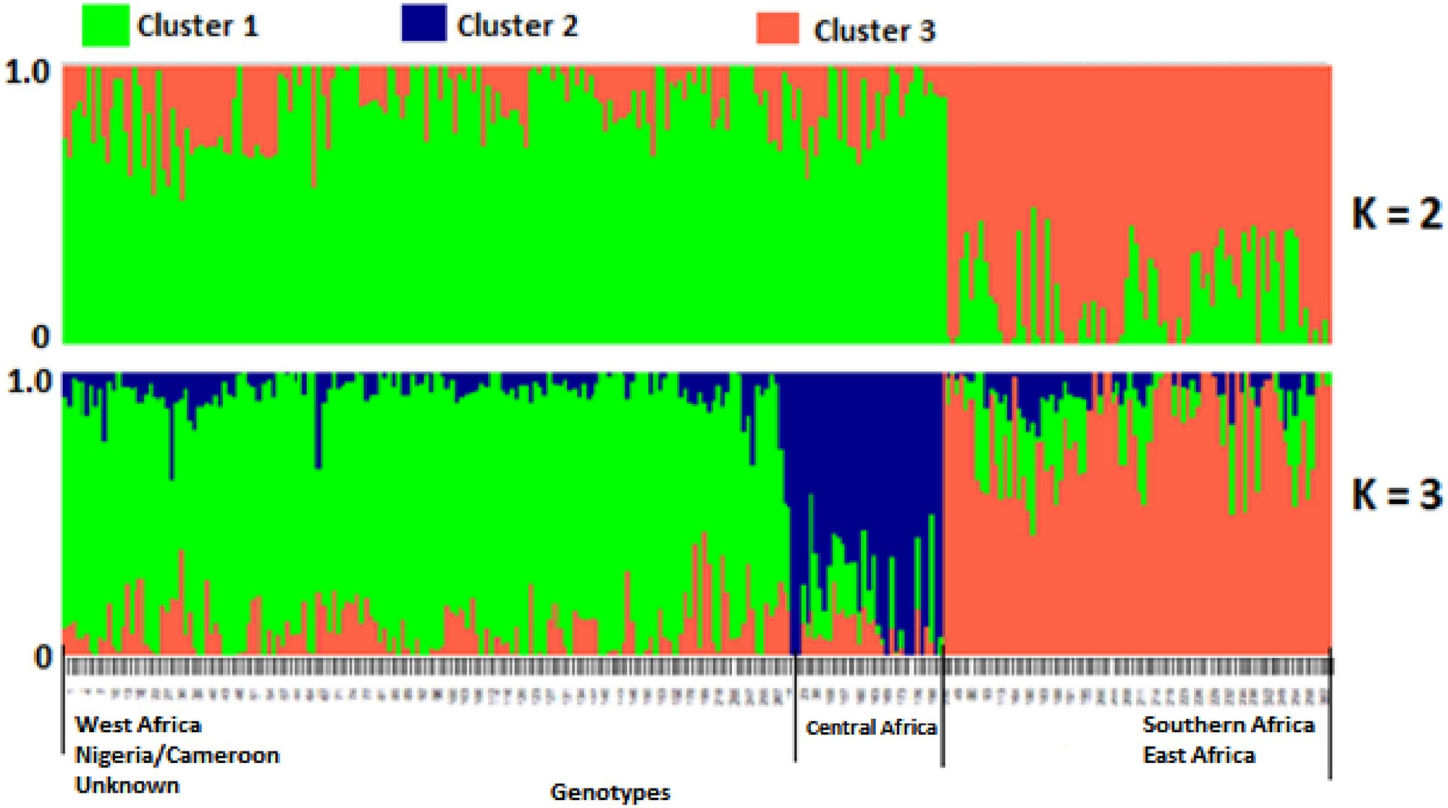
Population structure generated by ADMIXTURE model among 270 bambara groundnut genotypes (K = 2 top and K = 3 bottom). Each vertical bar represents one genotype that is partitioned in to up to K colored segments.

The admixture model-based clustering analysis approach was followed to investigate the population structure of 270 bambara groundnut accessions. The optimum cluster was three (K = 3) based on the model with the lower value of BIC preferred (S1 Fig). The three subpopulations mostly overlap with the geographic origin or the source of the accessions. The genotypes from Western Africa, Nigeria/Cameroon and the unknown origin belong to one subpopulation, which is the largest group. However, the Central Africa accessions belongs to subpopulation two, which is the smallest group, while the Southern and Eastern Africa accessions were in subpopulation three (Fig 6, S4 Table).

### Phenotypic variation in bambara groundnut germplasm

**Fig 7:**
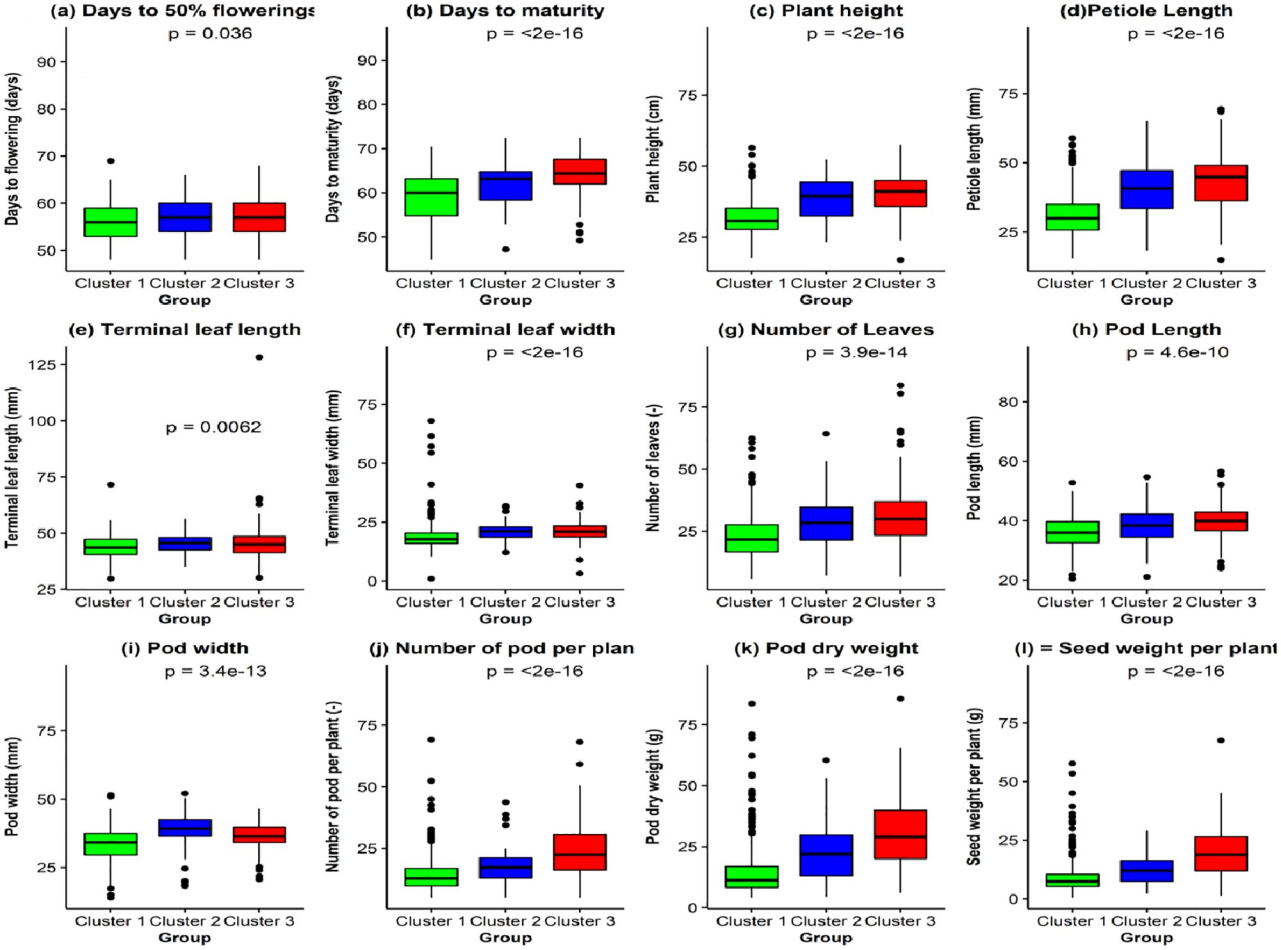
Boxplot showing the performances of bambara groundnut accessions in the identified three subpopulations for the studied morphological traits. Dots above and below boxplots are outliers, and lower and upper tails represent minimum and maximum values, respectively.

Highly significant (p < 0.001) genetic variation was observed among all morphological traits in the studied accessions (Table 5). Among the studied traits, days to maturity that ranged from 112–181 days, while other traits that exhibited greater ranges within the accessions includes number of leaves (22–318), petiole length (56.80–147.30), number of pod per plant (5.00–69.20), pod dry weight (4.10–85.70) and seed weight per plant (0.51–67.56). Furthermore, the traits that showed > 25% phenotypic coefficient of variation includes terminal leaf width (27.1%), petiole length (28.74%), number of leaves (41.99%), number of pods per plant (53.39%), pod dry weight (68.55%) and seed dry weight (74.57%). Genotypic coefficient of variation showed similar result trend for the traits number of leaves (29.71%), number of pods per plant (43.05%), pod dry weight (56.37%) and seed dry weight (60.12%). The average performance of the subpopulation showed that cluster three displayed the highest values for days to flowering, days to maturity, plant height, petiole length, number of leaves, pod length, number of pods per plant, pod dry weight and seed weight per plant (Fig 7). On the other hand, cluster two had the highest values for pod width, terminal leaf length and terminal leaf width, while cluster one showed the lowest value for all the studied morphological traits.

**Table 5:** Descriptive statistic and the morphological trait variation of the bambara groundnut accessions.

Traits	Min	Max	SD	Mean	GCV (%)	PCV (%)	F test
Days to 50% flowering	48.00	69.00	2.46	56.53	3.63	7.15	***
Days to maturity	112.00	181.00	6.51	152.28	6.92	9.19	***
Plant height (cm)	8.50	28.74	1.28	17.64	19.46	22.01	***
Petiole length (mm)	42.50	198.00	10.79	103.36	24.66	28.74	***
Terminal leaf length (mm)	41.60	92.00	4.10	62.38	7.17	11.74	***
Terminal leaf Width (mm)	9.00	68.00	3.06	19.95	16.18	27.08	***
Number of leaves	22.32	318.00	20.97	99.93	29.71	41.99	***
Pod length (mm)	12.80	48.00	1.93	23.42	11.65	16.47	***
Pod width (mm)	5.00	18.60	1.15	12.49	11.74	17.55	***
Number of pod per plant	5.00	69.20	4.02	18.01	43.05	53.39	***
Pod dry weight (g)	4.10	85.70	5.71	20.70	56.37	68.54	***
Seed weight per plant (g)	0.51	67.56	4.07	13.06	60.12	74.57	***

Min = minimum, Max = maximun, SD = standard deviation, GCV = genotypic coefficient of variation, PCV = phenotypic coefficient of variation.

## Discussion

### Genetic diversity and differentiation of the population

Understanding the genetic diversity and population structure of bambara groundnut landraces originating from various geographical regions of Africa is vital towards its conservation and utilization in developing methods to attain lucrative divergence in the breeding programs of this crop. The genetic diversity in germplasm plays an important role in the proficient investigation of useful alleles existing in landraces and diverse genotypes [52]. The higher genetic diversity found in the West Africa accessions (Hs = 0.476, Ht = 0.482) when compared with the other regions, the level of diversity which was not also much different from the Nigeria/Cameroon accessions (Hs = 0.478, Ht = 0.479), revealed that bambara groundnut might have originated from this region or there is relatively high evolutionary potential of bambara groundnut in this region, which could have been achieved through effective conservation of the crop in its habitat by the growers. Furthermore, there is a rich pool of diversity for bambara germplasm collected from these regions for continuous selection of adapted accessions for further improvement or selection of superior accessions for hybridization programme. This result is in accordance with reports based on agronomic and molecular markers [4,14,27,28]. Somta et al. [27] have reported comparable higher number of outcrossing rates that promote high genetic diversity in the West African region accessions than the other regions. In another study, Molosiwa et al. [14] found more molecular variation among the West African Bambara groundnut landraces compared to the Southern African landraces. The greater the genetic diversity of the germplasm, the higher is the chance of success in breeding some desirable traits. Higher genetic diversity obtained among the African accession obtained could be as a result that most bambara accessions are still landraces and have not received any significant improvement. Landraces are genetically heterogeneous [53] and are considered to be significantly more genetically diverse than cultivars because they are characterized of high magnitude of allelic and genetic diversity [54]. Furthermore, larger and older populations tends to have higher genetic diversity than small and newly established populations due to higher levels of accumulated and maintained genetic variation [55]. Hence, the mean value obtained on total gene diversity was moderate showing the suitability of DArT SNP marker in bambara groundnut. This implies the appropriateness of the marker type to characterize the accessions on genetic-molecular basis and could be further used for studying genetic linkage and QTL mapping of desirable traits, and consequently marker assisted selection (MAS) for improvement of bambara groundnut. Molosiwa et al. [14] and Olukola et al. [4] used DArT markers to distinguished bambara groundnut landraces collected from different populations; while Ho et al. [36] used the same marker to detect QTL region for internode length in this crop. Generally, low genetic distance was obtained among African bambara groundnut accessions and this suggests that bambara groundnut is a noncentric or oligocentric crop. The Nigeria/Cameroon region that had lowest genetic distance to West Africa region is because of their proximity to each other or their close relationship between the two populations. This report is in accordance with Rungnoi et al. [28]. Furthermore, lower genetic distances observed between Nigeria/Cameroon-West Africa accessions (0.002), East-Southern Africa accessions (0.003) showed that these populations shared many common alleles and are closely related. This result is in agreement with the report of Rungnoi et al. [28] and Somta et al. [27]. It was fitting to note that the unknown origin (sourced from United Kingdom) accessions showed high and distinct genetic distance with the accessions from the rest of the regions. The maximum genetic distance was also found between the unknown origin (sourced from United Kingdom) and the Southern African accessions. Such distant genetic relatedness has important implications in parental selection for the genetic improvement of the crop breeding program. Narvel et al. [56] reported that hybridization of parental lines selected from high divergence germplasm groups can result in high genetic recombination in the progenies that might be useful in enhancing the genetic gain from selection in the crop. The unknown origin (sourced from United Kingdom) accessions that showed the lowest genetic distance with the West African accessions suggested that these accessions are likely from West Africa origin. Somta et al. [27] used bambara groundnut accessions collected from Thailand and reported that it originated from both West Africa and East Africa.

The high percentage value of genetic diversity within-population obtained from AMOVA could be as a result of natural adaptation or extensive exchange of seeds among farmers between environments or because of common origin of the population, which might have led to bambara growers using the same seed continuously, without new introductions. In Ghana, seed sources for planting bambara groundnut are from farmer-saved seeds, exchange and market purchase [57]. This is likely to result a heterogeneous population of landraces, and hence, higher intra-landrace diversity, as against the homogenous population that would be expected, due to the autogamous breeding system of the crop [58]. Pasquet et al. [59] reported high intra population genetic diversity among the domesticated landraces of bambara groundnut. Low genetic variation among regions revealed lower migration of bambara groundnut landraces and selection of specific agronomic trait by the farmers that promotes local adaptation and genetic drift. Similar observation has been reported by Odongo et al. [9] and Ntundu et al. [26].

### Population structure and relationship of the bambara groundnut population

The cluster dendogram using a neighbor joining tree based on the geographical distribution of accessions showed that most of the bambara groundnut accessions from the same region did not cluster accurately based on their origin or regions. This could be due to low genetic differentiation among the populations, which suggests that the genetic background of bambara groundnut populations does not always correlate with their geographical origin or regions. Although, all the accessions of the same origin did not cluster according to their genetic background but majority of the accessions from the same origin clustered together which provides evidence in the origin and relationship of bambara groundnut landraces. Similar patterns of grouping of landraces according to geographical origin have been reported using morphological markers [58,60] and molecular markers [9,27,28] for collections of bambara groundnut landraces within countries and among regions. The clustering of some accessions from the different region into the same cluster might indicate the degree of relatedness between accessions from different regions, partly attributed to the transfer and or exchange of seeds between regions through gene banks and human migration.

Population structure analysis gives better understanding for genetic diversity and will help towards association mapping studies [61]. It also provides further information for selecting genetically divergent accessions for future hybridization programs [4]. The model-based population structure analysis grouped the population into three subgroups which overlapped mostly with the accessions geographic origin or the source of the genotypes. There is a clear separation between landraces from West Africa and Southern Africa, which reveals that bambara groundnut germplasm had more than one subpopulation. There has been argument that there could be more than a single centre of diversity and/or domestication of this species [14,21]. The grouping of Eastern and Southern Africa populations together indicates that bambara groundnut from this region could have originated from a common genetic background. Based on the population structure and neighbor-joining analysis, our data suggested that the unknown origin (United Kingdom sourced) accessions might have originated from Western Africa region, because they did not form a unique group, rather they clustered consistently with the subpopulation consisting of Western Africa accessions. This result suggests that bambara groundnut accessions (unknown origin sourced from United Kingdom) used in the study is from West Africa and might have be introduced through routes like trade and human migration. Molosiwa et al. [14] and Somta et al. [27] have used phylogenic analysis to reveal the origin of bambara groundnut accessions collected from Indonesia and Thailand, respectively. In one of this study, Molosiwa et al. [14] revealed that bambara groundnut accessions collected from Indonesia were originally from Southern Africa.

The differences observed in the morphological traits of the accessions in each cluster/group could be due to the genetic make-up or the influence of environment on the adaptation of the crop or selection of specific traits by farmers. Ntundu et al. [60] and Molosiwa et al. [14] have reported various phenotypic ranges for the studied quantitative traits. Cluster three that exhibited the highest value for most of the morphological traits might be used for selection of adapted genotypes for yield and other agronomic traits in this agro-ecology. The accessions from cluster one with the lowest value of days to maturity could serve as source of gene pool for early maturity for future breeding programme in bambara groundnut. Best parental lines that might be used by hybridization programs to improve early maturity and yield potential for areas with short growing period or prone to drought stress could be selected from clusters three and one.

## Conclusion

The results obtained from the present study indicated that DArT SNP marker is informative and selective and it might be widely used for molecular analysis of bambara groundnut. DArT SNP marker based on molecular characterization of bambara groundnut landraces from different regions revealed that variation exists among the accessions and the pattern of the genetic diversity varied across the five regions. Three main subpopulations of bambara groundnut germplasm was identified. Consequently, this result will contribute substantially to the management, conservation and association mapping and marker assisted selection of bambara groundnut accession for future improvement.

## Supporting information

S1 Fig Optimum number of K for the 270 bambara groundnut accessions. (PDF)

S1 Table Names of genotypes, countries and region of origin. (DOCX)

S2 Table Characteristic of the 3343 DArT SNP markers used for the analysis. (XLSX)

S3 Table Genetic distance matrix of the 270 accessions based on the DArT SNP marker. (XLSX)

S4 Table Number and percentage of bambara groundnut accessions assigned into the three selected clusters. (DOCX)
