# Supplementary figures and images for: Genetic diversity and population structure analysis of bambara groundnut (Vigna subterrenea L) landraces using DArT SNP markers
Source: PLoS One. 2021 Jul 1;16(7):e0253600. doi: 10.1371/journal.pone.0253600 (PMC8248626; doi:10.1371/journal.pone.0253600)

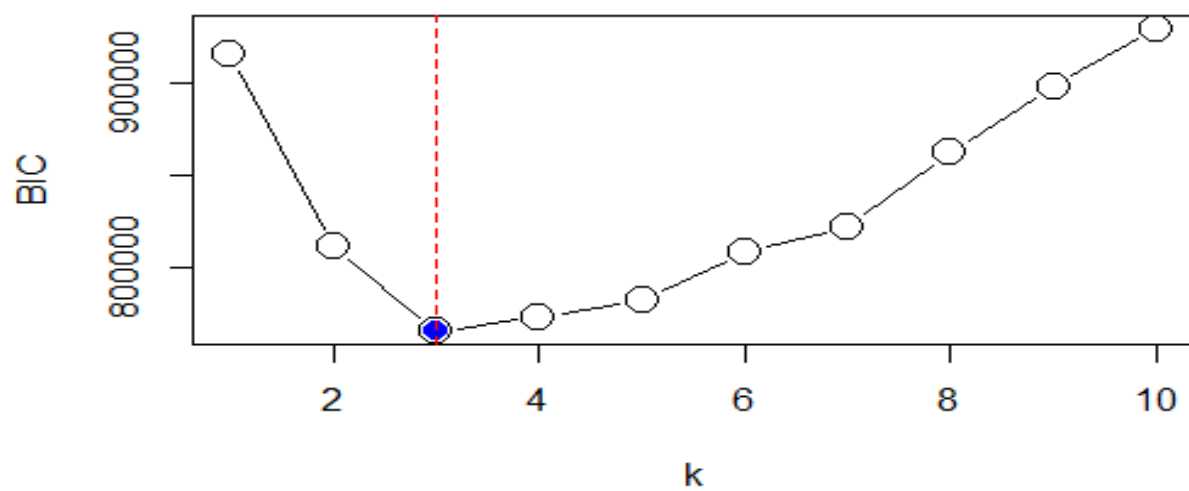

**S1 Fig: Optimum number of K for the 270 bambara groundnut accessions**

Supplement: S1 Fig — (PDF) [file pone.0253600.s001.pdf]
