# Supplementary material for: Genetic diversity and population structure analysis of bambara groundnut (Vigna subterrenea L) landraces using DArT SNP markers
Source: PLoS One. 2021 Jul 1;16(7):e0253600. doi: 10.1371/journal.pone.0253600 (PMC8248626; doi:10.1371/journal.pone.0253600)
Supplement: S1 Table — (DOCX) [file pone.0253600.s002.docx]

**S1 Table: Names of genotypes, countries and region of origin**

| **S/N** | **Genotype Name** | **Country** | **Region** | **Code** | **S/N** | **Genotype Name** | **Country** | **Region** | **Code** |
| --- | --- | --- | --- | --- | --- | --- | --- | --- | --- |
| 1 | TVSu-88 | Mali | West Africa | W | 136 | TVSu-182 | Nigeria | Nigeria/Cameroon | N/C |
| 2 | TVSu-565 | Cameroon | Nigeria/Cameroon | N/C | 137 | TVSu-1971 | Swaziland | Southern Africa | S |
| 3 | TVSu-2007 | DRC | Central Africa | C | 138 | TVSu-1383 | Togo | West Africa | W |
| 4 | MADURA AMPHETA | Malawi | Southern Africa | S | 139 | TVSu-1628 | Togo | West Africa | W |
| 5 | TVSu-235 | Ghana | West Africa | W | 140 | TVSu-893 | Zambia | Southern Africa | S |
| 6 | TVSu-305 | Burkina-Faso | West Africa | W | 141 | TVSu-1411 | Togo | West Africa | W |
| 7 | TVSu-412 | Cameroon | Nigeria/Cameroon | N/C | 142 | TVSu-377 | Tanzania | East Africa | E |
| 8 | TVSu-792 | Kenya | East Africa | E | 143 | TVSu-1860 | Zimbabwe | Southern Africa | S |
| 9 | TVSu-1161 | Burkina-Faso | West Africa | W | 144 | TVSu-226 | Ghana | West Africa | W |
| 10 | TVSu-1381 | Unknown origin sourced from United Kingdom | Unknown | UK | 145 | TVSu-247 | Gambia | West Africa | W |
| 11 | TVSu-1457 | Ghana | West Africa | W | 146 | TVSu-297 | Burkina-Faso | West Africa | W |
| 12 | TVSu-1797 | Malawi | Southern Africa | S | 147 | TVSu-1449 | Ghana | West Africa | W |
| 13 | TVSu-93 | Mali | West Africa | W | 148 | TVSu-1832 | Niger | West Africa | W |
| 14 | TVSu-1844 | Niger | West Africa | W | 149 | TVSu-1981 | DRC | Central Africa | C |
| 15 | TVSu-1610 | Togo | West Africa | W | 150 | TVSu-138 | Ghana | West Africa | W |
| 16 | TVSu-358 | Gambia | West Africa | W | 151 | TVSu-1630 | Togo | West Africa | W |
| 17 | TVSu-460 | Cameroon | Nigeria/Cameroon | N/C | 152 | TVSu-877 | Zambia | Southern Africa | S |
| 18 | TVSu-810 | Madagascar | East Africa | E | 153 | TVSu-1405 | Togo | West Africa | W |
| 19 | TVSu-2008 | DRC | Central Africa | C | 154 | TVSu-378 | Tanzania | East Africa | E |
| 20 | TVSu-1364 | CAR | Central Africa | C | 155 | TVSu-186 | Benin | West Africa | W |
| 21 | TVSu-1668 | Senegal | West Africa | W | 156 | TVSu-236 | Ghana | West Africa | W |
| 22 | TVSu-1799 | Malawi | Southern Africa | S | 157 | TVSu-248 | Gambia | West Africa | W |
| 23 | TVSu-100 | Mali | West Africa | W | 158 | TVSu-294 | Burkina-Faso | West Africa | W |
| 24 | TVSu-1887 | Tanzania | East Africa | E | 159 | TVSu-1453 | Ghana | West Africa | W |
| 25 | KAYERA | Malawi | Southern Africa | S | 160 | TVSu-1834 | Niger | West Africa | W |
| 26 | TVSu-1607 | Togo | West Africa | W | 161 | TVSu-200 | Benin | West Africa | W |
| 27 | TVSu-345 | Gambia | West Africa | W | 162 | TVSu-1363 | CAR | Central Africa | C |
| 28 | TVSu-600 | Nigeria | Nigeria/Cameroon | N/C | 163 | TVSu-1632 | Togo | West Africa | W |
| 29 | TVSu-870 | Zambia | Southern Africa | S | 164 | TVSu-1404 | Togo | West Africa | W |
| 30 | TVSu-1289 | CAR | Central Africa | C | 165 | TVSu-386 | Tanzania | East Africa | E |
| 31 | TVSu-1378 | CAR | Central Africa | C | 166 | TVSu-1851 | Zimbabwe | Southern Africa | S |
| 32 | TVSu-1700 | Togo | West Africa | W | 167 | TVSu-239 | Ghana | West Africa | W |
| 33 | TVSu-1803 | Cameroon | Nigeria/Cameroon | N/C | 168 | TVSu-249 | Gambia | West Africa | W |
| 34 | TVSu-101 | Mali | West Africa | W | 169 | TVSu-295 | Burkina-Faso | West Africa | W |
| 35 | TVSu-1892 | Botswana | Southern Africa | S | 170 | TVSu-1458 | Ghana | West Africa | W |
| 36 | MAKATA | Malawi | Southern Africa | S | 171 | TVSu-1837 | Niger | West Africa | W |
| 37 | TVSu-1480 | Ghana | West Africa | W | 172 | TVSu-2022 | Burundi | Central Africa | C |
| 38 | TVSu-272 | Nigeria | Nigeria/Cameroon | N/C | 173 | TVSu-1362 | CAR | Central Africa | C |
| 39 | TVSu-369 | Tanzania | East Africa | E | 174 | TVSu-1648 | Senegal | West Africa | W |
| 40 | TVSu-671 | Nigeria | Nigeria/Cameroon | N/C | 175 | TVSu-1403 | Togo | West Africa | W |
| 41 | TVSu-1034 | Zimbawe | Southern Africa | S | 176 | TVSu-387 | Tanzania | East Africa | E |
| 42 | TVSu-1308 | CAR | Central Africa | C | 177 | TVSu-1843 | Niger | West Africa | W |
| 43 | TVSu-1379 | Unknown origin sourced from United Kingdom | Unknown | UK | 178 | TVSu-240 | Ghana | West Africa | W |
| 44 | TVSu-1737 | Zambia | Southern Africa | S | 179 | TVSu-250 | Gambia | West Africa | W |
| 45 | TVSu-1813 | Cameroon | Nigeria/Cameroon | N/C | 180 | TVSu-287 | Nigeria | Nigeria/Cameroon | N/C |
| 46 | TVSu-145 | Ghana | West Africa | W | 181 | TVSu-1464 | Ghana | West Africa | W |
| 47 | KADZIWUNDE | Malawi | Southern Africa | S | 182 | TVSu-1839 | Niger | West Africa | W |
| 48 | TVSu-1474 | Ghana | West Africa | W | 183 | TVSu-1667 | Senegal | West Africa | W |
| 49 | TVSu-280 | Nigeria | Nigeria/Cameroon | N/C | 184 | TVSu-203 | Benin | West Africa | W |
| 50 | TVSu-376 | Tanzania | East Africa | E | 185 | TVSu-1181 | Burkina-Faso | West Africa | W |
| 51 | TVSu-515 | Cameroon | Nigeria/Cameroon | N/C | 186 | TVSu-1295 | CAR | Central Africa | C |
| 52 | TVSu-1312 | CAR | Central Africa | C | 187 | TVSu-1165 | Burkina Faso | West Africa | W |
| 53 | TVSu-1382 | Togo | West Africa | W | 188 | TVSu-1101 | Zimbabwe | Southern Africa | S |
| 54 | TVSu-1742 | Zambia | Southern Africa | S | 189 | TVSu-1294 | CAR | Central Africa | C |
| 55 | TVSu-1821 | Cameroon | Nigeria/Cameroon | N/C | 190 | CREAM | Malawi | Southern Africa | S |
| 56 | TVSu-160 | Ghana | West Africa | W | 191 | TVSu-762 | Zambia | Southern Africa | S |
| 57 | TVSu-1959 | Zimbabwe | Southern Africa | S | 192 | TVSu-132 | Ghana | West Africa | W |
| 58 | MUSANDIONE KUDA | Malawi | Southern Africa | S | 193 | TVSu-99 | Mali | West Africa | W |
| 59 | TVSu-1473 | Ghana | West Africa | W | 194 | TVSu-1775 | Malawi | Southern Africa | S |
| 60 | TVSu-289 | Benin | West Africa | W | 195 | MASO AMARIA | Malawi | Southern Africa | S |
| 61 | TVSu-379 | Tanzania | East Africa | E | 196 | TVSu-1651 | Senegal | West Africa | W |
| 62 | TVSu-688 | Zambia | Southern Africa | S | 197 | TVSu-1164 | Burkina-Faso | West Africa | W |
| 63 | TVSu-902 | Zambia | Southern Africa | S | 198 | TVSu-1666 | Senegal | West Africa | W |
| 64 | TVSu-1316 | CAR | Central Africa | C | 199 | TVSu-1176 | Burkina-Faso | West Africa | W |
| 65 | TVSu-1380 | Unknown origin sourced from United Kingdom | Unknown | UK | 200 | TVSu-1186 | Burkina-Faso | West Africa | W |
| 66 | TVSu-1756 | Malawi | Southern Africa | S | 201 | TVSu-691 | Zambia | Southern Africa | S |
| 67 | TVSu-1830 | Mali | West Africa | W | 202 | TVSu-381 | Tanzania | East Africa | E |
| 68 | TVSu-193 | Benin | West Africa | W | 203 | TVSu-1788 | Malawi | Southern Africa | S |
| 69 | TVSu-1998 | DRC | Central Africa | C | 204 | TVSu-393 | Sudan | East Africa | E |
| 70 | CHIKOPE CHA NYANI | Malawi | Southern Africa | S | 205 | TVSu-776 | Zambia | Southern Africa | S |
| 71 | TVSu-290 | Benin | West Africa | W | 206 | TVSu-984 | Zimbabwe | Southern Africa | S |
| 72 | TVSu-383 | Tanzania | East Africa | E | 207 | TVSu-1111 | Zimbabwe | Southern Africa | S |
| 73 | TVSu-704 | Zambia | Southern Africa | S | 208 | TVSu-1828 | Mali | West Africa | W |
| 74 | TVSu-1146 | Guinea | West Africa | W | 209 | TVSu-1018 | Zimbabwe | Southern Africa | S |
| 75 | TVSu-1327 | CAR | Central Africa | C | 210 | TVSu-12 | Nigeria | Nigeria/Cameroon | N/C |
| 76 | TVSu-1407 | Togo | West Africa | W | 211 | TVSu-702 | Zambia | Southern Africa | S |
| 77 | TVSu-1773 | Malawi | Southern Africa | S | 212 | TVSu-2012 | DRC | Central Africa | C |
| 78 | TVSu-1831 | Mali | West Africa | W | 213 | TVSu-1301 | CAR | Central Africa | C |
| 79 | TVSu-194 | Benin | West Africa | W | 214 | TVSu-402 | Cameroon | Nigeria/Cameroon | N/C |
| 80 | TVSu-1999 | CAR | Central Africa | C | 215 | TVSu-833 | Madagascar | East Africa | E |
| 81 | TVSu-299 | Burkina-Faso | West Africa | W | 216 | TVSu-939 | Zambia | Southern Africa | S |
| 82 | TVSu-388 | Sudan | North Africa | N | 217 | TVSu-1112 | Zimbabwe | Southern Africa | S |
| 83 | TVSu-770 | Zambia | Southern Africa | S | 218 | TVSu-1838 | Niger | West Africa | W |
| 84 | TVSu-1147 | Guinea | West Africa | W | 219 | TVSu-1042 | Zimbabwe | Southern Africa | S |
| 85 | TVSu-1340 | CAR | Central Africa | C | 220 | TVSu-1202 | Burkina-Faso | West Africa | W |
| 86 | TVSu-1633 | Togo | West Africa | W | 221 | TVSu-709 | Zambia | Southern Africa | S |
| 87 | TVSu-1792 | Malawi | Southern Africa | S | 222 | TVSu-2014 | DRC | Central Africa | C |
| 88 | TVSu-541 | Cameroon | Nigeria/Cameroon | N/C | 223 | TVSu-1305 | CAR | Central Africa | C |
| 89 | TVSu-1922 | Senegal | West Africa | W | 224 | TVSu-431 | Cameroon | Nigeria/Cameroon | N/C |
| 90 | TVSu-1840 | Niger | West Africa | W | 225 | TVSu-838 | Nigeria | Nigeria/Cameroon | N/C |
| 91 | TVSu-1324 | CAR | Central Africa | C | 226 | TVSu-926 | Zambia | Southern Africa | S |
| 92 | TVSu-1649 | Senegal | West Africa | W | 227 | TVSu-1115 | Zimbabwe | Southern Africa | S |
| 93 | TVSu-1419 | Togo | West Africa | W | 228 | TVSu-1841 | Niger | West Africa | W |
| 94 | TVSu-341 | Nigeria | Nigeria/Cameroon | N/C | 229 | TVSu-597 | Nigeria | Nigeria/Cameroon | N/C |
| 95 | TVSu-1902 | Malawi | Southern Africa | S | 230 | TVSu-1056 | Zimbabwe | Southern Africa | S |
| 96 | TVSu-206 | Benin | West Africa | W | 231 | TVSu-1237 | Nigeria | Nigeria/Cameroon | N/C |
| 97 | TVSu-242 | Gambia | West Africa | W | 232 | TVSu-723 | Zambia | Southern Africa | S |
| 98 | TVSu-251 | Gambia | West Africa | W | 233 | TVSu-2071 | Nigeria | Nigeria/Cameroon | N/C |
| 99 | TVSu-273 | Nigeria | Nigeria/Cameroon | N/C | 234 | TVSu-1306 | CAR | Central Africa | C |
| 100 | TVSu-1466 | Ghana | West Africa | W | 235 | TVSu-441 | Cameroon | Nigeria/Cameroon | N/C |
| 101 | TVSu-1930 | Malawi | Southern Africa | S | 236 | TVSu-1663 | Senegal | West Africa | W |
| 102 | TVSu-1842 | Niger | West Africa | W | 237 | TVSu-903 | Zambia | Southern Africa | S |
| 103 | TVSu-1321 | CAR | Central Africa | C | 238 | TVSu-1139 | Guinea | West Africa | W |
| 104 | TVSu-1653 | Senegal | West Africa | W | 239 | TVSu-1655 | Senegal | West Africa | W |
| 105 | TVSu-1418 | Togo | West Africa | W | 240 | TVSu-869 | Zambia | Southern Africa | S |
| 106 | TVSu-353 | Nigeria | Nigeria/Cameroon | N/C | 241 | TVSu-106 | Mali | West Africa | W |
| 107 | TVSu-189 | Benin | West Africa | W | 242 | TVSu-1245 | Nigeria | Nigeria/Cameroon | N/C |
| 108 | TVSu-211 | Ghana | West Africa | W | 243 | TVSu-725 | Zambia | Southern Africa | S |
| 109 | TVSu-244 | Gambia | West Africa | W | 244 | TVSu-2074 | Nigeria | Nigeria/Cameroon | N/C |
| 110 | TVSu-252 | Gambia | West Africa | W | 245 | TVSu-1307 | CAR | Central Africa | C |
| 111 | TVSu-254 | Nigeria | Nigeria/Cameroon | N/C | 246 | TVSu-519 | Cameroon | Nigeria/Cameroon | N/C |
| 112 | TVSu-1467 | Ghana | West Africa | W | 247 | TVSu-1661 | Senegal | West Africa | W |
| 113 | TVSu-1954 | Zimbabwe | Southern Africa | S | 248 | TVSu-681 | Zambia | Southern Africa | S |
| 114 | TVSu-1396 | Togo | West Africa | W | 249 | TVSu-115 | Cote d'Ivoire | West Africa | W |
| 115 | TVSu-1611 | Togo | West Africa | W | 250 | TVSu-1251 | Nigeria | Nigeria/Cameroon | N/C |
| 116 | TVSu-901 | Zambia | Southern Africa | S | 251 | TVSu-304 | Burkina Faso | West Africa | W |
| 117 | TVSu-1417 | Togo | West Africa | W | 252 | TVSu-1093 | Zimbabwe | Southern Africa | S |
| 118 | TVSu-364 | Nigeria | Nigeria/Cameroon | N/C | 253 | TVSu-127 | Nigeria | Nigeria/Cameroon | N/C |
| 119 | TVSu-1872 | Zimbabwe | Southern Africa | S | 254 | TVSu-730 | Zambia | Southern Africa | S |
| 120 | TVSu-216 | Ghana | West Africa | W | 255 | TVSu-2076 | Nigeria | Nigeria/Cameroon | N/C |
| 121 | TVSu-245 | Nigeria | Nigeria/Cameroon | N/C | 256 | TVSu-1315 | CAR | Central Africa | C |
| 122 | TVSu-303 | Burkina-Faso | West Africa | W | 257 | TVSu-527 | Cameroon | Nigeria/Cameroon | N/C |
| 123 | TVSu-1428 | Togo | West Africa | W | 258 | TVSu-1753 | Malawi | Southern Africa | S |
| 124 | TVSu-1798 | Malawi | Southern Africa | S | 259 | TVSu-677 | Zambia | Southern Africa | S |
| 125 | TVSu-1964 | Swaziland | Southern Africa | S | 260 | TVSu-1163 | Burkina-Faso | West Africa | W |
| 126 | TVSu-1392 | Togo | West Africa | W | 261 | TVSu-1182 | Burkina-Faso | West Africa | W |
| 127 | TVSu-1617 | Togo | West Africa | W | 262 | TVSu-335 | Nigeria | Nigeria/Cameroon | N/C |
| 128 | TVSu-90 | Mali | West Africa | W | 263 | TVSu-1098 | Zimbabwe | Southern Africa | S |
| 129 | TVSu-1414 | Togo | West Africa | W | 264 | TVSu-1291 | CAR | Central Africa | C |
| 130 | TVSu-368 | Nigeria | Nigeria/Cameroon | N/C | 265 | TVSu-742 | Zambia | Southern Africa | S |
| 131 | TVSu-1865 | Zimbabwe | Southern Africa | S | 266 | TVSu-752 | Zambia | Southern Africa | S |
| 132 | TVSu-217 | Nigeria | Nigeria/Cameroon | N/C | 267 | TVSu-1319 | CAR | Central Africa | C |
| 133 | TVSu-246 | Ghana | West Africa | W | 268 | TVSu-987 | Zimbabwe | Southern Africa | S |
| 134 | TVSu-302 | Burkina-Faso | West Africa | W | 269 | TVSu-1771 | Malawi | Southern Africa | S |
| 135 | TVSu-1447 | Ghana | West Africa | W | 270 | TVSu-637 | Nigeria | Nigeria/Cameroon | N/C |
