# Supplementary material for: Genetic diversity and population structure analysis of bambara groundnut (Vigna subterrenea L) landraces using DArT SNP markers
Source: PLoS One. 2021 Jul 1;16(7):e0253600. doi: 10.1371/journal.pone.0253600 (PMC8248626; doi:10.1371/journal.pone.0253600)
Supplement: S4 Table — (DOCX) [file pone.0253600.s005.docx]

**S4 Table: Number and percentage of bambara groundnut accessions assigned into the three selected clusters**

|  |  | **K = 3** |  |  |  |
| --- | --- | --- | --- | --- | --- |
| **REGION** | **Number of Genotypes** | **Cluster 1** | **Cluster 2** | **Cluster 3** | **Admixture** |
| Nigeria/Cameroon | 39 | 27 | 4 | 2 | 6 |
| West Africa | 117 | 99 | 5 | 9 | 4 |
| Central Africa | 29 | 3 | 18 | 6 | 2 |
| Southern Africa | 67 | 8 | 1 | 50 | 8 |
| East Africa | 15 | 2 | 1 | 9 | 3 |
| Unknown origin | 3 | 3 | 0 | 0 | 0 |
| Percentage |  | 52.59 | 10.74 | 28.15 | 8.52 |
